# In vivo anti-malarial activity of the hydroalcoholic extract of rhizomes of *Kniphofia foliosa* and its constituents

**DOI:** 10.1186/s12936-020-03552-7

**Published:** 2021-01-01

**Authors:** Yonatan Alebachew, Daniel Bisrat, Solomon Tadesse, Kaleab Asres

**Affiliations:** Department of Pharmaceutical Chemistry and Pharmacognosy, School of Pharmacy, College of Health Sciences, Addis Ababa University, Addis Ababa, Ethiopia

**Keywords:** *Kniphofia foliosa*, Antimalarial activity, In vivo, Phenolic fractions, *Plasmodium berghei*, Knipholone, Dianellin

## Abstract

**Background:**

*Kniphofia foliosa* is a flamboyant robust perennial herb which has dense clumps and tick upright rhizomes with leaves at the base. In Ethiopia, it has several vernacular names including *Abelbila*, *Ashenda*, *Amelmela*, *Yeznjero Ageda*, *Shemetmetie* and *Yezinjero Ageda*. The plant is endemic to Ethiopian highlands, where its rhizomes are traditionally used for the treatment of malaria, abdominal cramps and wound healing. In the present study, the 80% methanol extract of *K. foliosa* rhizomes and its constituents are tested against *Plasmodium berghei *in mice**.**

**Methods:**

Isolation was carried out using column and preparative thin layer chromatography (PTLC). The chemical structures of the compounds were elucidated by spectroscopic methods (ESI–MS, 1D and 2D-NMR). Peters’ 4-day suppressive test against *P. berghei *in mice was utilized for in vivo anti-malarial evaluation of the test substances.

**Results:**

Two compounds, namely knipholone and dianellin were isolated from the 80% methanolic extract of *K. foliosa* rhizomes, and characterized. The hydroalcoholic extract (400 mg/kg) and knipholone (200 mg/kg) showed the highest activity with chemosuppression values of 61.52 and 60.16%, respectively. From the dose–response plot, the median effective (ED_50_) doses of knipholone and dianellin were determined to be 81.25 and 92.31 mg/kg, respectively. Molecular docking study revealed that knipholone had a strong binding affinity to *Plasmodium falciparum* l-lactate dehydrogenase (pfLDH) target.

**Conclusion:**

Results of the current study support the traditional use of the plant for the treatment of malaria.

## Background

Malaria is one of the most serious life-threatening infectious diseases [1]. It occurs mostly in poor tropical and subtropical areas of the world, where the Africa region accounted for 93% of all malaria cases and 94% of malaria deaths [2]*.* Most often, pregnant women and children under five years old are severely affected [3]. For instance, from the total deaths due to malaria in 2018, 67% or 272,000 were children under 5 years of age. That is nearly 745 children under age 5 daily or one child under five every two minutes dies of malaria in 2018 alone and most of these deaths occurred in sub-Saharan Africa [2]. In addition to funding shortfalls and fragile health systems, the major contributor to malarial morbidity and mortality is almost certainly the increasing resistance of malaria parasites to available drugs [4].

In Ethiopia, there has been success in the past recent years to reduce malaria burden. However, it is still prevalent in 75% of the country putting over 45 million people at risk [5]. The disease accounts for 7% of outpatient visits to health clinics and represents the third largest cause of morbidity [6]. In addition, 8% of global *Plasmodium vivax* malaria cases occur in Ethiopia [2]. Hence, the fight against malaria in Ethiopia remains a public health priority.

Natural products from plants have played a huge role throughout history in the fight against malaria. For instance, the aqueous extracts of cinchona bark were an effective anti-malarial preparation for more than 300 years [7]. Later, quinine, the major active alkaloid of cinchona was isolated in the 1820s. Similarly, artemisinin was isolated from the cold ether extracts of the leaves of Chinese traditional medicinal herb, *Artemisia annua* in 1972 [8]. Inspired by these, different classes of anti-malarial compounds were isolated from a variety of plant families. Although hundreds of potent anti-malarial compounds were isolated from African traditional medicine, there has not been any clinically successful molecule [9–11]*.*

The genus *Kniphofia* belongs to the family Asphodelaceae which comprises 70 species mainly confined to Africa [12]. Fifteen of these species have been recorded in Eastern Africa, of which five including *Kniphofia foliosa* are endemic to Ethiopia [13, 14]. The rhizomes of *K. foliosa* are traditionally used for the treatment of abdominal cramps, malaria and wounds [15]. Previously, six in vitro active anti-malarial compounds were isolated from roots of *K. foliosa* by Wube et al*.* [16] and Abdissa et al*.* [17]. In continuation of search for lead anti-malarial compounds from Ethiopian medicinal plants [18–20], the in vivo anti-malarial activity of the rhizomes of *K. foliosa* and its constituents are investigated.

## Methods

### Chemicals and instruments

Chromatographic separations were performed by analytical TLC on Silica gel 60 F254 (0.2 mm thick), Silica gel 60 (0.063–0.200 mm) for column chromatography (70–230 mesh) (Merck KGaA, Darmstadt, Germany) and solid phase separation on Isolute C_18_ columns (10 g; IST, Hengoed, UK). Trisodium citrate was obtained from BDH Chemicals Ltd, England, Giemsa was purchased from ESJAY Chemicals, Maharashtra 401,504, India, and pure chloroquine phosphate was supplied by Ethiopian Pharmaceutical Manufacturing Factory (EPHARM, Ethiopia).

NMR spectra were recorded at 500 MHz for ^1^H and 125 MHz for ^13^C on a Bruker Avance DMX400 FT-NMR spectrometer (Bruker, Billerica, Massachusetts, USA) using tetramethylsilane (TMS) as internal standard. All spectra were measured in CDCl_3_, except for compound **1**, which was dissolved in CD_3_OD. HRMS were determined on a Shimadzu LC‐MS Advanced spectrometer (Shimadzu, Kyoto, Japan) in the positive and negative modes.

### Plant material

The rhizomes of *K. foliosa* were collected in February 2017 from Mount Kundi near the city of Ankober in Shewa region of Central Ethiopia and identified by Professor Sebsibe Demisew at the National Herbarium, Addis Ababa University (AAU), Addis Ababa, Ethiopia, where voucher specimens were deposited (Collection number: YA01/2017).

### Experimental animals

Healthy, 5–6 weeks old Swiss albino mice weighing 20–25 g were employed throughout the experiment. Female mice were used for acute toxicity study as per the OECD Guideline 425 [21], and either male or female mice were employed for the anti-malarial activity tests [22, 23]. All mice used for the experiments were obtained either from the animal house of the School of Pharmacy (SoP), AAU or purchased from the Ethiopian Health Nutrition and Research Institute (EHNRI). The animals were maintained under natural lighting conditions (12 h light and 12 h dark cycle) at room temperature and relative humidity of approximately 50%. They were provided with food and water ad libitum and acclimatized for one week before the commencement of the experiment.

### Rodent parasite

Chloroquine (CQ) sensitive ANKA strain of *Plasmodium berghei* was used. The donor mice infected with the parasite was obtained from the EHNRI. The parasites were maintained by serial blood passage from mouse to mouse at 5 days interval. All procedures followed were in accordance with the Guide for the Care and Use of Laboratory Animals [24] and were approved by the Institutional Review Board of the SoP, College of Health Sciences, AAU.

### Extraction, fractionation and isolation

The air-dried powdered rhizomes of *K. foliosa* were soaked in 80% methanol at room temperature for 4 days with occasional shaking. Removal of the organic solvent using rotary evaporator (BUCHI Rotavapor™ R-300, Switzerland) followed by freeze drying of the remaining water extract yielded a dark red gummy solid. Portion of the dried extract was dissolved in 5% KOH solution and partitioned with chloroform to remove the nonphenolic components. The aqueous phase was acidified with 2% HCl and then further partitioned with chloroform. The chloroform layer was collected and concentrated in a rotary evaporator to give a dried solid designated crude phenolic fraction I. The reddish solid mass (methanol soluble) formed between the acidified aqueous and chloroform layers was collected as phenolic fraction II. Purification of phenolic fraction I by preparative TLC (Additional file 1: Fig. S1) gave KFP-1 (18 mg, 0.048%). Furthermore, portion of the hydroalcoholic extract was fractionated on silicagel flash column chromatography to yield three fractions. Fraction 1 was eluted with 100% chloroform, fractions 2 and 3 with a mixture of chloroform and methanol (1:1), and fraction 4 with 100% methanol. Fraction 3 was concentrated and freeze dried to give viscous solid, which was further purified by sequential PTLC and solid phase extraction on Isolute C_18_ columns to give YKFM-2 (14 mg, 0.04%) (Additional file 1: Fig. S2).

### Acute oral toxicity testing

Acute oral toxicity study was conducted as per the internationally accepted protocol of OECD Guideline 425 [21]. Fifteen healthy Swiss female albino mice weighing 20–25 g were randomly grouped into 3 each having 5 mice. Following 3–4 h of fasting (food only), one mouse from each group was orally administered 2000 mg/kg of the hydroalcoholic extract, KFP-1 and YKFM-2, consecutively. This was repeated on the remaining mice for the following four days. The mice were then observed individually for any physical or behavioural changes, such as loss of appetite, ruffled fur, lacrimation, mortality, and other signs of toxicity for 4 h. The same procedure was followed for the remaining mice for the next five consecutive days and the results recorded. The follow-up observations were continued for all mice for 14 days.

### In vivo anti-malarial assay

#### Inoculation

Blood smear was prepared on microscope slides from blood films taken from the donor (infected) mouse tail. The smear was fixed with methanol and stained with Giemsa to count the parasitaemia of the donor under a microscope (Primo Star, Carl Zeiss, Germany). The mice were then inoculated on day 0 with parasitized erythrocytes obtained from the donor by cardiac puncture using a sterile syringe when the parasitaemia level was 30–40%. Blood from the donor was collected on a Petri dish containing 2% trisodium citrate and was immediately diluted with uninfected mouse blood and normal saline in such a way that the final volume contains 5 × 10^7^ infected erythrocytes/ml of blood. The diluted blood (0.2 ml) was injected into all the experimental mice intraperitoneally (i.p) [25, 26].

### 4-Day suppressive test

The standard 4-day suppressive method was used for anti-malarial evaluation of the test substances [27]. The test was carried out in two phases. The extract and phenol fractions were evaluated in the first phase followed by KFP-1 and YKFM-2 in the second phase. Doses were selected based on the acute toxicity results whereby the middle dose was taken as one tenth of the limit dose which is 200 mg/kg. The higher dose was twice (400 mg/kg) the middle dose and the lower dose was one half (100 mg/kg) of the middle dose [28]. During the first phase, 60 inoculated mice were randomly grouped into 12 groups each having five mice. Groups 1 served as a negative control (distilled water, Vehicle1, 0.2 ml) for the extract and phenolic fraction 2 treated groups, while group 2 animals were used as a negative control (1% tween 80, Vehicle2, 0.2 ml) for phenolic fraction 1 treated group. The third group which served as a positive control was treated with standard pure chloroquine (25 mg/kg/day). The remaining nine groups were treatment groups and received 100, 200, and 400 mg/kg/day of the hydroalcoholic extract and the two phenol fractions. The dosage regimen for the pure compounds was chosen from the preliminary test results obtained from experiments carried out on a few mice. Similarly, during evaluation of KPF-1 and YKFM-2, 45 inoculated mice were randomly grouped into 9 groups, each containing five mice. The first two groups were negative controls (distilled water, Vehicle3, 0.2 ml) and positive controls (standard pure chloroquine, 25 mg/kg/day). The rest of the groups were treatment groups and received KFP-1 (25, 50, 100 and 200 mg/kg/day) and YKFM-2 (25, 50, 100 mg/kg/day). All the test substances were administered orally using oral gavage. Treatment was started 3 h post-infection (p.i) on day 0 and continued daily for the next 3 days (i.e. from day 0 to day 3). On the fifth day (or day 4), two Giemsa-stained blood smears were prepared from each mice to count the number of parasites under the microscope (Primo Star, Carl Zeiss, Germany) with an oil immersion objective of 100× magnification power [21, 23, 29].

Mean percent parasitaemia and percent suppression were calculated using the following formulae.

$${\text{\% Parasitaemia }} = \left( {\frac{{\text{Number of parasitized RBC}}}{{\text{Total number of RBC count}}}} \right) \times 100$$

$${\text{\% Suppression }} = \left( {\frac{{{\text{Mean parasitaemia of negative control }}{-}{\text{ Mean parasitaemia of treated}}}}{{\text{Mean parasitaemia of negative control }}}} \right)\times {100}$$

### Body weight and survival time measurements

Body weight of each mouse was measured on day 0 before infection and on day 4. Survival time was recorded from day 1 to day 28 post inoculation. Then, the mean body weight and mean survival time were calculated for each group [20].

### Molecular docking study

Docking study was carried out on two crystal structures of *Plasmodium* enzymes plasmepsin II (Protein Data Bank; PDB: 4CKU) and l-lactate dehydrogenase (pfLDH) [PDB: 1LDG], using SeeSar10.0 software (BioSolveIT, Sankt Augustin, Germany). For plasmepsin II, the selected binding site was the binding pocket of a previously designed inhibitor P2FE-400, 5-(1,1-dioxido-1,2-thiazinan-2-yl)-N1-((2S,3R)-3-hydroxy-4-((2-(3-methoxyphenyl)propan-2-yl)amino)-1-phenylbutan-2-yl)-N3,N3-dipropylisophthalamide; while for pfLDH, the cofactor nicotinamide adenine dinucleotide (NADH) binding site was selected for docking. The HYDE score was used to estimate the binding affinity of the molecules [30, 31].

### Statistical analysis

Data analysis was carried out using IBM SPSS (Statistical Package for Social Sciences) Statistics for Windows, Version 20.0. Armonk, NY: IBM Corp. Results were expressed as mean ± standard error of mean (M ± SEM). The statistical significance was determined by one-way ANOVA followed by Tukey post hoc test to compare percent suppression (activity), mean survival time and percent changes in body weight of the *P. berghei* infected mice among the treatment and control groups. *P* < 0.05 was considered significant.

## Results and discussion

### Structural elucidation of the isolated compounds

Phytochemical investigation of the rhizome extract of *K. folosia* over silica gel column and PTLC resulted in the isolation of two compounds. Compound **1** (YKFM-2): R_f_: 0.69 (a mixture of EtOAc and *n*-butanol-acetic acid–water, upper phase; 4:1:5 in a ratio of 1:1); compound **2** (KFP-1) R_f_: 0.47 (toluene/EtOAc; 5:1).

**Fig. 1:**
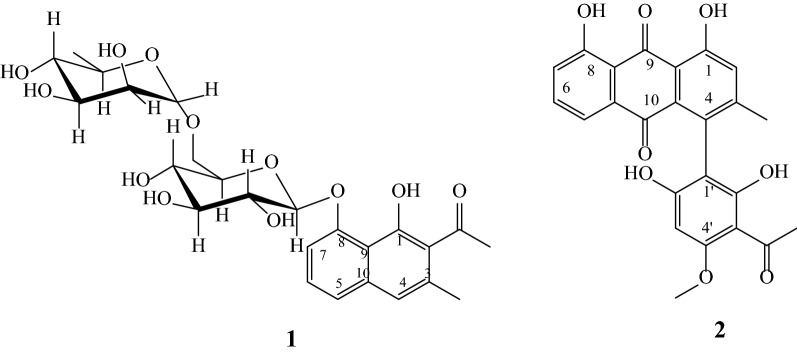
Chemical structures of dianellin (**1**) and knipholone (**2**)

Compound **1** was obtained as a light red amorphous solid. The positive high resolution-ESI mass spectrum gave a pseudomolecular ion at *m/z* 547.1619 [M + Na]^+^ (calcd. *m/z* 547.1791 [M + Na]^+^, corresponding to a molecular formula C_25_H_32_O_12_. In the ^1^H NMR spectrum, four proton signals which resonated at δ 7.12 (*s*, H-4), 7.28 (1H, *dd*, *J* = 1.3, 7.3 Hz, H-7), 7.36 (1H, *d*, *J* = 8.2 Hz, H-6) and 7.40 (1H, *dd*, *J* = 1.3, 8.2 Hz, H-5) indicated the presence of aromatic ring moiety. Three of these proton signals which are multiplets imply that they are found in close proximity (or are adjacent) and the fourth singlet aromatic proton peak at δ 7.12 (*s*, H-4) provides clues for the presence of a fused aromatic ring system. The presence of a disaccharide unit in compound **1** was revealed by the typical anomeric proton signals at δ 3.31 (1H, *m*, H-4′′) and 5.05 (1H, *d*, *J* = 7.9 Hz, H-1′). The proton peaks from δ 5.05 to 2.91 further justify the presence of a disaccharide moiety. The ^13^C spectrum region from δ 76.82 to 66.59 also confirmed that the compound contains a disaccharide moiety. In addition, the two elevated ^13^C sugar signals at δ 102.84 and 100.86 indicate that the sugar units are linked through acetal bond. Furthermore, the absence of one CH signal in the sugar region (δ 76.82–δ 66.59) suggests one of the sugar units to be rhamnose. And this was found to be in good agreement with ^13^C NMR reports of similar glycosides [32, 33]. Hence, the disaccharide moiety was confirmed to be rhamnose-glucose 1,6 linkage. In addition, the presence of 10 ^13^C signals from δ 154.71 to δ 110.5 implies that the fused aromatic ring system is naphthalene. Six of these carbon signals are absent from DEPT spectrum indicating they are quaternary aromatic carbons. Besides, two of them are elevated (δ 154.71 and δ 151.49) suggesting that they are oxygenated quaternary aromatic carbons. On the other hand, the two less elevated (δ 136.74 and δ 113.54) quaternary aromatic carbons are the bridgehead carbons of the fused aromatic system [33]. The remaining two quaternary aromatic carbon signals resonated at δ 124.73 (C-2) and δ 133.30 (C-3). Lastly, the ^13^C signals at 207.7 and 41.3 are the carbonyl carbon and its acetyl methyl. Therefore, based on the above evidence and in comparison with ^1^H and ^13^C NMR data of the same and related compounds [33, 34], the structure of compound **1** was determined to be dianellin or 1-(1-hydroxy-3-methyl-8-(((2S,3R,4S,5S,6R)3,4,5-trihydroxy-6-((((2S,3S,4S,5S,6R)3,4,5-trihydroxy-6-methyltetrahydro-2H-pyran-2-yl)oxy)methyl)tetrahydro-2H-pyran-2yl)oxy)naphthalen-2-yl) ethanone (Fig. 1). Table 1 summarizes the NMR data of compound **1**.

**Table 1:** ^1^H and ^13^C NMR data of compound **1** measured in methanol-*d*_4_

Present data			Reference data [33]	
Position	δ_C_ (ppm)	δ_H_ (ppm)	δ_C_ (ppm)	δ_H_ (ppm)
1	151.49	–	150.2	–
2	124.73	–	125.2	–
3	133.30	–	132.8	–
4	119.58	7.12 (1H, *s*)	119.4	7.21 (1H, *s*)
5	122.58	7.40 (1H, *dd*, *J* = 1.3, 8.2 Hz)	122.3	7.47 (1H, *dd*, *J* = 1.0, 8.0 Hz)
6	127.20	7.36 (1H, *d*, *J* = 8.2 Hz)	127.3	7.40 (1H, *dd*, *J* = 8.0, 8.0 Hz)
7	110.51	7.28 (1H, *dd*, *J* = 1.3, 7.3, Hz)	110.7	7.30 (1H, *dd*, *J* = 1.0, 8.0 Hz)
8	154.71	–	154.2	–
9	113.54	–	113.2	–
10	136.74	–	135.7	–
1′	102.84	5.05 (1H, *d*, *J* = 7.9 Hz)	102.6	5.04 (1H, *d*, *J* = 7.5 Hz)
2′	73.57	3.46 (1H, *t*, *J* = 8.8 Hz)	73.3	3.39 (1H, *m*)
3′	76.82	3.37 (1H, *m*)	76.2	3.36 (1H, *m*)
4′	70.13	2.91 (1H, *m*)	70.1	3.18 (1H, *m*)
5′	76.10	3.68 (1H, *m*)	76.0	3.59 (1H, *m*)
6′	66.59	4.05 ( 1H, *d*, *J* = 8.9 Hz); 3.63 (1H, *m*)	66.6	3.93 (1H, *dd*, *J* = 1.5, 11.0 Hz); 3.50 (2H, *m*)
1′′	100.86	4.71 (1H, *d*, *J* = 1.4 Hz)	100.7	4.62 (1H, *d*, *J* = 1.5 Hz)
2′′	70.84	3.84 (1H, *dd*, *J* = 1.6, 3.4 Hz)	70.4	3.68 (1H, *m*)
3′′	71.03	3.63 (1H, *m*)	70.7	3.50 (2H, *m*)
4′′	72.59	3.31 (1H, *m*)	71.9	3.20 (1H, *m*)
5′′	68.55	3.52 (1H, *m*)	68.4	3.49 (1H, *m*)
6′′	16.55	1.17 (3H, *d*, *J* = 6.2 Hz)	17.7	1.12 (3H, *d*, *J* = 6.0 Hz)
ArCH_3_	18.49	2.25 (3H, *s*)	19.0	2.25 (3H, *s*)
COCH_3_	41.3	2.97 (3H, *s*)	31.9	2.52 (3H, *s*)
*C*OCH_3_	207.07	–	204.4	–

*s*, singlet, *d*, doublet, *dd*, doublet of doublets, *m*, multiplet, *br*, broad, *t*, triplet

Compound **2** was isolated as an orange colored amorphous solid. The molecular formula was determined to be C_24_H_18_O_8_ by the positive-ion ESIMS (*m/z* 458.21 [M + Na]^+^), which was also consistent with ^1^H and ^13^C NMR spectral data. The chelated hydroxyl protons shown as singlet peaks at δ 12.6 (1H*, s*, -OH, H-1) and 11.9 (1H*, s*, -OH, H-8) and the typical ABC pattern of the proton peaks at δ 7.57 (1H, *dd*, *J* = 8, 7 Hz, H-6), 7.55 (1H, *dd*, *J* = 7, 1.5 Hz, H-5), 7.21 (1H, *dd*, *J* = 8, 1.5 Hz, H-7) indicate the presence of chrysophanol moiety. Besides, the singlet aromatic proton signal present at δ 7.28 suggests that it is found adjacent to a substituted aromatic carbon. The ^13^C and DEPT spectra of compound **2** also support the presence of chrysophanol moiety [35, 36]. Moreover, from the ^13^C spectrum, additional aromatic carbon signals at δ 152.44, 132.72, 125.75, 125.31, and 120.11 together with the ^1^H peaks at 14.3 (*s*, OH), 6.19 (*s*, aromatic H), 5.7 (*s* (*br*), OH) and 3.91 (*s*, OCH_3_) indicate the attachment of a methyl etherified acetylphloroglucinol moiety to chrysophanol. These data in comparison with the reported ^1^H and ^13^C NMR results identify compound **2** as knipholone (Fig. 1). Table 2 summarizes the NMR data of compounds **2**.

**Table 2:** ^1^H and ^13^C NMR data of compounds **2** in chloroform-*d*

Present data				Reference data [35]	
Position	δ_C_ (ppm)	δ_H_ (ppm)		δ_C_ (ppm)	δ_H_ (ppm)
1	161.69	12.6 (1H*, s*, −OH)		161.7	12.53 (1H, *s*, −OH)
1a	115.22	–		114.7	–
2	125.31	7.28 (1H, *s*)		124.6	7.32 (1H, *q, J* = 0.7 Hz)
3	152.44	–		151.6	
4	125.75	–		128.5	
4a	132.72	–		131.6	
5	120.11	7.55 (1H, *dd*, *J* = 1.5, 7 Hz)		119.3	7.56 (1H, *dd*, *J* = 1.5, 7 Hz)
5a	134.27	–		134.4	
6	137.12	7.57 (1H, *dd*, *J* = 7, 8 Hz)		137.4	7.75 (1H, *dd*, *J* = 7, 8 Hz)
7	123.85	7.21 (1H, *dd*, *J* = 1.5, 8 Hz)		123.3	7.30 (1H, *dd*, *J* = 1.5, 8 Hz)
8	159.51	11.9 (1H, *s*, -OH)		161.1	12.0 (1H, *s*, -OH)
8a	115.37	–		115.5	–
9	192.68	–		192.5	–
10	182.66	–		181.9	–
1’	106.07	–		104.7	–
2’	163.27	5.7 (1H, *s* (*br*), -OH)		163.3	8.95 (1H, *s* (*br*), -OH)
3’	107.14	–		107.3	–
4’	163.07	–		162.4	–
5’	90.61	6.19 (1H, *s*)		91.2	6.24 (1H, *s*)
6’	162.85	14.3 (1H, *s*, -OH)		161.9	–
ArCH_3_	21.02	2..21 (3H, *s*)		20.4	2.17 (3H, *d*, *J* = 0.7 Hz)
OCH_3_	55.56	3.91 (3H, *s*)		55.6	3.98 (3H, *s*)
COCH_3_	33.14	2.70 (3H, *s*)		32.6	2.62 (3H, *s*)
COCH_3_	202.3	–		202.3	–

*s*, singlet, *d*, doublet, *dd*, doublet of doublets, *q*, quartet, *m*, multiplet, *br*, broad

### Acute oral toxicity

Acute oral toxicity test results of this study documented that the 80% methanol extract of *K. foliosa,* knipholone and dianellin were safe at a dose of 2000 mg/kg [21, 37]. After 72 h, the animals tolerated the administered dose although immediate mild toxicity signs such as ruffled fur, loss of appetite and slight sleepiness, which disappeared few hours after administration were observed. Also, there was no mortality within 14 days of observation which entails that the LD_50_s of the extract, knipholone and dianellin are above 2000 mg/kg.

### Anti-malarial activity of the hydroalcoholic extract

The 80% methanol extract of *K. folosia* showed chemosuppressive effect against *P. berghei* in mice (Table 3). At all dose levels tested, the extract exhibited a statistically significant (*p* < 0.001) dose dependent effect. The extract showed the highest activity with 61.52 and 51.39% suppression at 400 and 200 mg/kg, respectively. Moreover, at doses of 200 and 400 mg/kg, the extract significantly extended the survival days of treated groups compared to the negative controls, indicating that the extract has the capacity to lower the overall pathologic effect of the parasite in mice. However, there was no significant difference in percent change in weight before and after treatment among groups except with the positive control group. According to Deharo et al*.* [38], anti-malarial activity of the 80% methanol extract of *K. folosia* can be regarded as good since it showed greater than 50% suppression at a dose of 200 mg/kg. Previous studies demonstrated that medicinal plants rich in anthraquinones such as aloes and senna possess notable in vivo anti-malarial activity [39, 40].

**Table 3:** Anti-malarial activity of the 80% rhizome extract of *Kniphofia folosia* in mice infected with *Plasmodium berghei*

Test substances	Dose (mg/kg/day)	Percent parasitaemia	Percent suppression	Mean survival time (in days)
Vehicle1	0.2 ml	35.9860 ± 1.22034	0.0000	6.0000 ± 0.31623
KF100	100 mg	24.2100 ± 1.18037	32.7200^a*c**d*e*^	9.4000 ± 0.50990^a**e*^
KF200	200 mg	17.4920 ± 0.67964	51.3900^a*b**e*^	9.6000 ± 0.92736^a*e*^
KF400	400 mg	13.8480 ± 0.76024	61.5200^a*b*e*^	8.4000 ± 0.24495^e*^
Chloroquine	25 mg	0.0140 ± 0.00600	99.8000^a*^	27.2000 ± 0.58310^a*b*c*d*^

Values are presented as mean ± SEM; n = 5; ^a^compared to vehicle1 (distilled water), ^b^compared to KF100, ^c^compared to KF200, ^d^compared to KF400, ^e^compared to chloroquine; * (p < 0.001); **(p < 0.01); KF = 80% extracts of *K. folosia*, numbers refer to doses in mg/kg/day

### Anti-malarial activity of the phenol fractions and their constituents

**Fig. 2:**
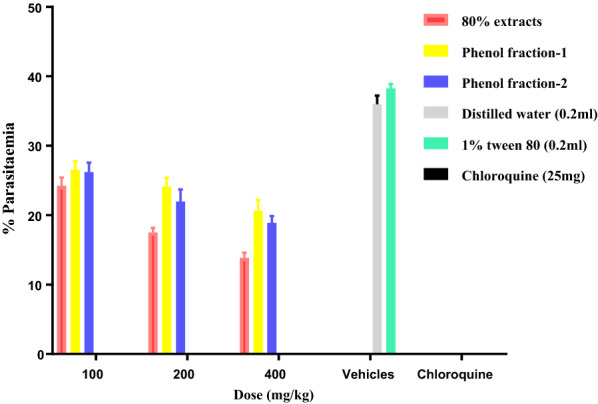
Anti-malarial activity of the 80% extract and phenol fractions of rhizomes of *Kniphofia folosia* in mice infected with *Plasmodium berghei* in 4-day suppression test*.* Values are presented as mean ± SEM; n = 5

The two phenolic fractions of *K. folosia* were also found to have activity against *P. berghei* in mice (Fig. 2). Compared to their respective negative controls, both factions possessed significant suppressive activity at all dose levels tested. They showed the highest activity at 400 mg/kg with fraction 1 and fraction 2 causing 46.32% and 47.53% suppression, respectively. Both fractions prolonged the mean survival days of the treatment groups by 2 days relative to their negative controls although it was not statistically significant. No significant difference in percent change in weight was noted in the treatment groups when compared with the positive controls. Therefore, it can be deduced that the phenolic fractions of *K. folosia* are moderate in their in vivo anti-malarial activity, congruent with earlier reports that extracts containing phenolic compounds and their glycosides have modest levels of antiplasmodial activity [41–43].

**Fig. 3:**
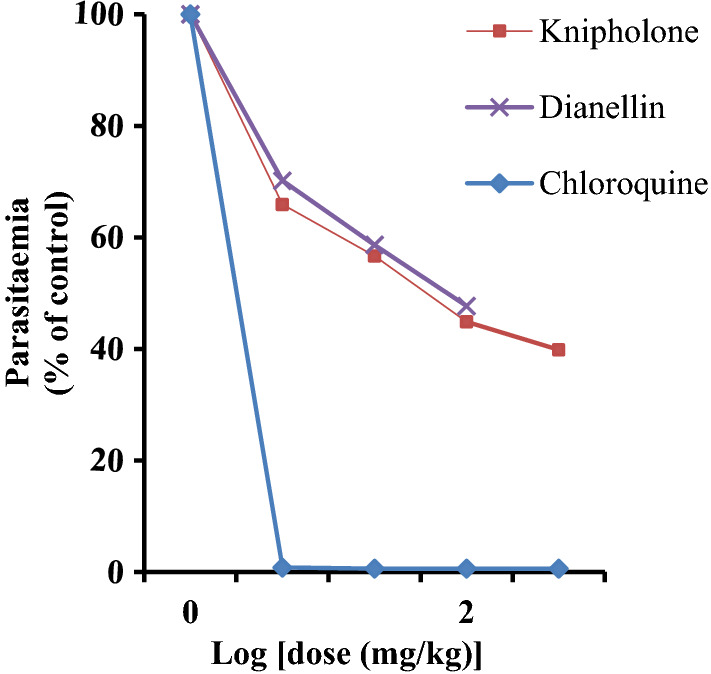
Anti-malarial activity of knipholone and dianellin in mice infected with *Plasmodium berghei.* The ED_50_ was estimated from a plot of log dose against parasitaemia (expressed as a percentage of the control). Values are presented as mean ± SEM; n = 5

Among the isolated compounds, knipholone displayed the strongest anti-malarial activity against *P. berghei* infected mice (Table 4). Although knipholone and dianellin showed significant suppression at all dose levels tested, the former displayed superior activity with percent suppression values of 55.14 and 60.16% at doses of 100 and 200 mg/kg, respectively. Moreover, it significantly prolonged the mean survival days of the treatment groups (Table 4). The dose–response plot (Fig. 3) disclosed that the ED_50_ values of knipholone and dianellin were 81.25 and 92.31 mg/kg, respectively. However, neither of the compounds caused significant difference in percent change of weight among the treated groups.

**Table 4:** Anti-malarial activity of knipholone and dianellin in mice infected with *Plasmodium berghei*

Test substances	Dose (mg/kg/day)	Percent parasitaemia	Percent suppression	Mean survival time (in days)
Vehicle3 Knipholone	0.2 ml	46.3560 ± 1.46925	0.0000	6.4000 ± 0.50990
	25 mg	30.5440 ± 1.45634	34.1200^a*d*e*h*i*^	8.8000 ± 0.37417^i*^
	50 mg	26.2640 ± 1.80001	42.6400^a*e**h*i*^	9.0000 ± 0.54772^i*^
	100 mg	20.7940 ± 0.91475	55.1400^a*b*e**f*g*i*^	9.2000 ± 0.73485^a***i*^
	200 mg	18.4680 ± 0.97391	60.1600^a*b*c**f*g*i*^	9.4000 ± 0.24495^a**i*^
Dianellin	25 mg	32.5280 ± 0.96771	29.8300^a*c*d*e*h*h*i*^	7.6000 ± 0.24495^i*^
	50 mg	25.9408 ± 0.77243	44.0400^a*d*e*g*h*i*^	8.2000 ± 0.37417^i*^
	100 mg	21.4303 ± 0.84156	53.7700^a*b*c*f*g*i*^	8.2000 ± 0.37417^i*^
Chloroquine	25 mg	0.0140 ± 0.00600	9.8000^a‒h*^	27.4000 ± 0.400000^a‒h*^

Values are presented as mean ± SEM; n = 5; ^a^compared to vehicle3 (distilled water), ^b^compared to knipholone 25 mg, ^c^compared to knipholone 50 mg, ^d^compared to knipholone 100 mg, ^e^compared to knipholone 200 mg, ^f^compared to dianellin 25 mg, ^g^compared to dianellin 50 mg, ^h^compared to dianellin 100 mg, ^i^compared to chloroquine; * (p < 0.001); **(p < 0.01); ***(p < 0.05); numbers refer to doses in mg/kg/day

Previously, the methanol and dichloromethane extracts of *K. folosia* as well as dimeric anthraquinones, knipholone and dianellin isolated therefrom have been shown to have in vitro activity against chloroquine-resistant (W2) and chloroquine-sensitive (D6 and 3D7) strains of *P. falciparum* [16, 17]. The ED_50_ of knipholone (1.49 μg/ml) against 3D7 strains [16] was much lower than that of dianellin which showed IC_50_ values of 3.14 and 5.47 µg/ml against W2 and D6 strains, respectively [17]. In addition, knipholone isolated from *Bulbine capitata* and *Bulbine frutescens* has been reported to possess in vitro activity against chloroquine-resistant (K1) and chloroquine-sensitive (NF54) strains with IC_50_ values of 1.06 and 1.7 µM, respectively [44, 45].

In this study, the acute toxicity as well as the in vivo antiplasmodial activities of the various extracts of *K. folosia* and its major constituents was evaluated against *P. berghei* in mice. Since members of the genus *Kniphofia* have been documented to produce anthraquinones and related phenols [46, 47], the total extract was further fractionated into phenolic and nonphenolic fractions. Results of the current study demonstrated that the hydroalcoholic extract, the fractions as well as the isolated compounds significantly suppressed parasitaemia indicating that they possesses blood schizonticidal activity on early infection of mice with *P. berghei*. The results also revealed that the in vivo ED_50_ values of knipholone (81.25 mg/kg) and dianellin (92.31 mg/kg) correlate well with their reported in vitro activities. After 4 days of treatment with different doses of knipholone and dianellin, there were significant differences in percent parasite suppression among the treatment groups. However, there was no significant change in mean survival time among mice administered with different doses of knipholone and dianellin except that the animals which received 100 and 200 mg/kg of knipholone survived longer than those given 25 mg/kg of the compound. This is an indication that the different doses of the test compounds have the same effect on overall pathologic effect of the parasite in mice concurrent with previous results obtained for the crude extract and solvent fractions of *Strychnos mitis* leaves [23]. To the best of our knowledge, this is the first report on acute toxicity and in vivo anti-malarial evaluation of *K. folosia* and its constituents.

Perusal of literature reveals that a number of promising anthraquinones and preanthraquinones leads such as visimione, rufigallol, uveoside, aloin and phenyl anthraquinones have been isolated and/or synthesized [16, 48–50]. These compounds are considered as oxidants like artemisinins and 4-aminoquinolines. More importantly, they are catalytic oxidants that enhance the production of reactive oxygen species (ROS) inside parasitized erythrocytes or increase these cells’ susceptibility to oxygen radicals. The free oxygen radicals formed interact with haem or other biomolecular targets inhibiting its tetramerization to the insoluble haemozoin (malaria pigment) [51, 52]. Knipholone, being an anthraquinone derivative, is anticipated to undergo one-electron oxidation and subsequently interact with haem (or other biomolecular targets) thereby inhibiting its tetramerization (or detoxification of haem). Similarly, because of the structural similarity of dianellin and phlorizin, a monoglucosidechalcone, its anti-malarial mechanism of action could be due to inhibition of the solute transporter of the host cell membrane induced by the parasite invasion [53, 54].

### Molecular docking study

To get further insight on the mechanism of action of the isolated compounds and to study their binding interaction and identify hypothetical binding motifs, a docking study of knipholone, dianellin and the standard anti-malarial drugs chloroquine and artemisinin were carried out on two crystal structures of target enzymes. The two *Plasmodium* enzymes were plasmepsin II (PDB code 4cku) involved in haemoglobin metabolism by the parasite, and *P. falciparum* l-lactate dehydrogenase (pfLDH) (PDB code 1ldg) involved in glycolysis (or glucose metabolism of the parasite) [55–57]. There is a strong suggestion that haemoglobin digesting enzymes found in the food vacuole of the *Plasmodium* and pfLDH are potential anti-malarial chemotherapeutic targets for chloroquine and related aminoquionlones, anthraquinones and other oxidative phenolic compounds [58–63]. Gossypol and other phenolic compounds were also found to be pfLDH inhibitors [64, 65]. Besides, chloroquine has been found to bind to the cofactor (NADH) binding site of pfLDH acting as a competitive inhibitor [66].

**Fig. 4:**
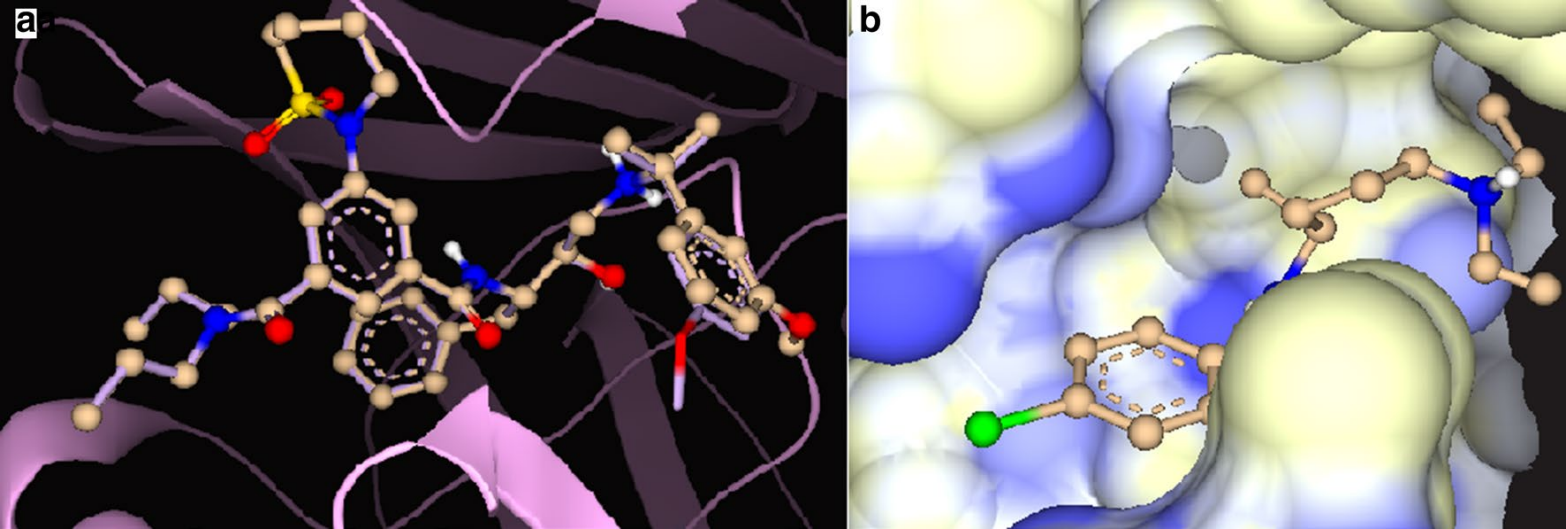
**a** Superimposition of redocked P2FE-400 (shown as a solid line) with its original position (shown in ball-stick model) as a complex (co-crystal) in the binding site of the crystal structure of plasmepsin II (PDB 4cku). **b** Surface representation showing chloroquine in the binding site of plasmepsin II with lipophilicity coloring, white representing hydrophobic pockets and blue representing hydrophilic pockets. Chloroquine is shown in ball-stick model

The binding modes of P2FE-400, a designed inhibitor of plasmepsin II, knipholone and chloroquine to plasmepsin II are shown in Fig. 4. P2FE-400 showed the highest and strongest affinity for the aspartic protease, plasmepsin II, with the HYDE score of − 38.3 kJ/mol. The aspartic protease plasmepsin II has two aspartic acid residues Asp34 and Asp214 (the catalytic dyad) that serve as proton donors and acceptors, respectively, in the amide hydrolysis of peptide bonds in proteins. As shown in the current study and also described by Jaudzems et al. [67], P2FE-400 forms four hydrogen bonds with the catalytic dyad (Asp34 and Asp214), Val78 and Ser218 amino acid residues. Chloroquine showed a comparable binding affinity with an estimated HYDE score of − 19.7 kJ/mol. The Cl substituent of chloroquine was found to be unsuitable for binding in the hydrophobic cavity of plasmepsin II. Chloroquine forms hydrogen bonds with Gly36 and Val78 amino acid residues. Knipholone and dianellin showed weak binding interaction with HYDE score of − 6.0 and − 4.2 kJ/mol, respectively. Nonetheless, knipholone forms two hydrogen bonds with one of the catalytic dyad (Asp214) and Val78 amino acid residues.

**Fig. 5:**
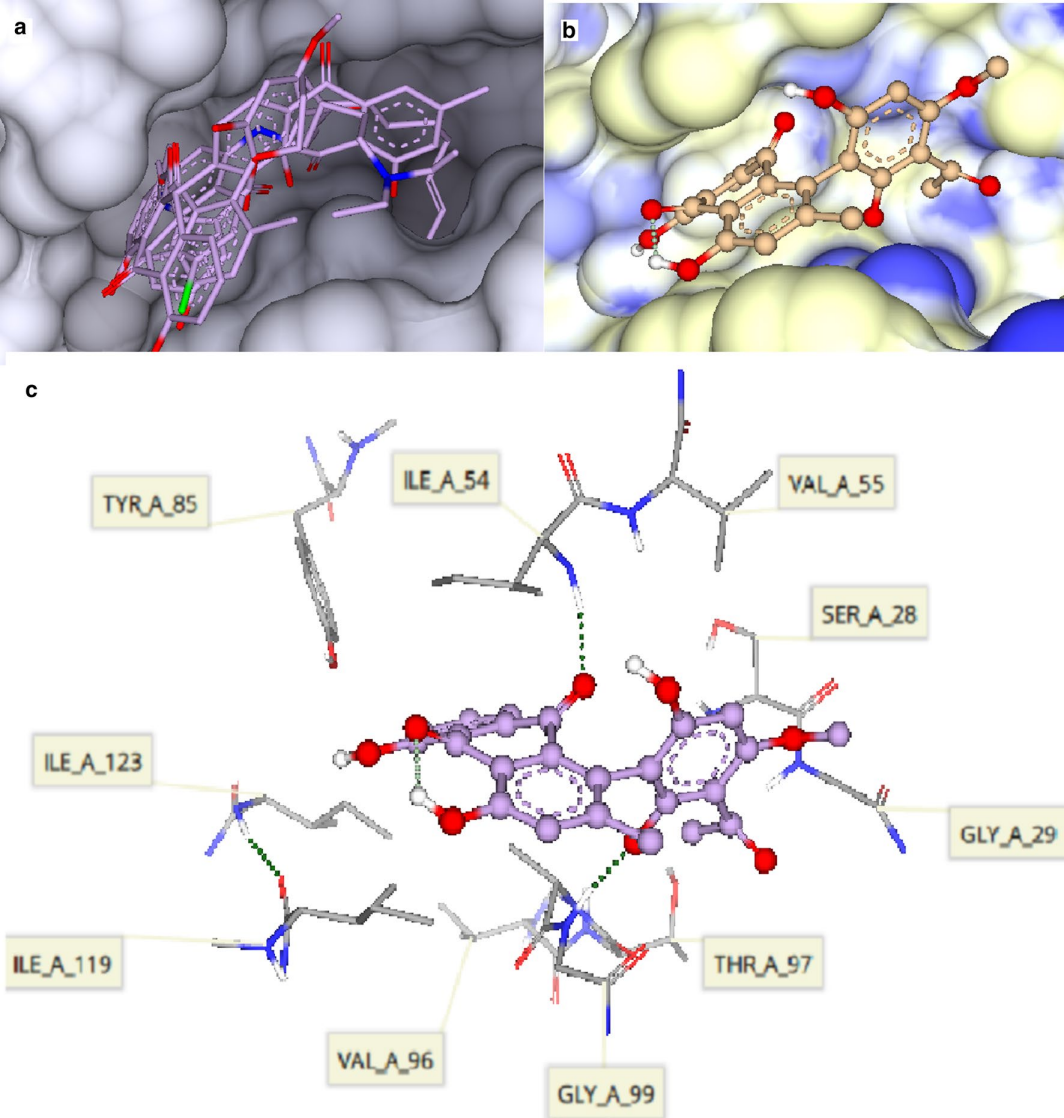
**a** Surface representation showing the superimposed compounds in the binding site of *Plasmodium falciparum* l-lactate dehydrogenase (pfLDH) (PDB1ldg). **b** Surface representation showing knipholone in the binding site of pfLDH with lipophilicity coloring, white representing hydrophobic pockets and blue representing hydrophilic pockets. Knipholone is shown in ball-stick model. **c** Binding interaction of knipholone with amino acid residues of pfLDH

The binding modes of knipholone and chloroquine to pfLDH binding site are shown in Fig. 5. Knipholone (− 29.1 kJ/mol) showed stronger binding interaction with pfLDH than chloroquine (− 24.7 kJ/mol). Knipholone forms hydrogen bonds with Ile54 and Val98 amino acid residues. Its carbonyl oxygen (at C-9) and hydroxyl group in ring A (at C-1) of the anthraquinone moiety, and the carbonyl oxygen (at C-3′) of the phloroglucinol moiety together with the *ortho* and *para* hydroxyl groups (at C-1′ and C-4′) are not favourable for binding. From the experimental data, there were seven hydrogen bonds in pfLDH-NADH complex, of which four are observed in this study [68]. Chloroquine on its part showed two hydrogen bonds with Asp53 and Gly99 amino acid residues. One of the N-ethyl groups of chloroquine is not needed in the hydrophilic binding sites. Moreover, the actual pfLDH-chloroquine complex also showed two hydrogen bonds with Glu122 and Gly99 [66]. In contrast, dianellin did not show binding interaction with pfLDH.

## Conclusion

In conclusion, the rhizome extracts of *K. folosia* possess in vivo anti-malarial effect against *P. berghei* in mice. This finding in conjunction with the safety profile obtained from the acute oral toxicity results support the traditional claim of the plant for the treatment of malaria. The current molecular docking study also identified the binding motifs of the isolated compounds showing that knipholone interact with important amino acid residues in the binding site of the target enzymes.

## Supplementary Information

**Additional file 1: Fig. S1.** Isolation protocol of knipholone (**2**). **Fig. S2.** Isolation protocol of dianellin (**1**). **Fig. S3.** I: ^1^H, ^13^C, DEPT and HRMS of YKFM-2 (Dianellin). **Fig. S4.** II: ^1^H, ^13^C, DEPT and HRMS of KFP-1 (Knipholone). **Table S1.** Antimalarial activity of the phenolic fractions of *Kniphofia folosia* in mice infected with *Plasmodium berghei*. **Table S2.** Prediction of partition coefficient Log P, aqueous solubility Log S and partition coefficient for partially dissociated compounds Log D of the compounds. **Table S3.** Docking result of compounds on the crystal structure of plasmepsin II (4cku) and plasmodium falciparum l-lactate dehydrogenase (pfLDH) (PDB 1ldg). **Table S4.** Acute oral toxicity results of dianellin. **Fig. S4.** Microscope slide photos of the negative control groups (A and B), dianellin and knipholone treated groups (C-E) and positive control group (F).

## Data Availability

The following are given in the supplementary figures or tables: Isolation protocols of compounds **1** and** 2** and their NMR and HRMS spectra; Summaries of anti-malarial activity of the phenolic fractions along with acute toxicity results of one of the isolated compounds; Predicted physicochemical properties of the standard drugs, isolated compounds and P2FE-400; Summaries of the docking results; and Microscope slide photos.

## Abbreviations

AAU: Addis Ababa University
EHNRI: Ethiopian Health Nutrition and Research Institute
ED_50_: Median effective dose
i.p: Intraperitoneally
NADH: Nicotinamide adenine dinucleotide
pfLDH: *Plasmodium falciparum* L-lactate dehydrogenase
p.i: Post-infection
PTLC: Preparative thin layer chromatography
PDB: Protein Data Bank
SoP: School of Pharmacy
