# Supplementary material for: In vivo anti-malarial activity of the hydroalcoholic extract of rhizomes of Kniphofia foliosa and its constituents
Source: Malar J. 2021 Jan 1;20:3. doi: 10.1186/s12936-020-03552-7 (PMC7777528; doi:10.1186/s12936-020-03552-7)
Supplement: Supplementary file 1 — Additional file 1: Fig. S1. Isolation protocol of knipholone (2). Fig. S2. Isolation protocol of dianellin (1). Fig. S3. I: 1H, 13C, DEPT and HRMS of YKFM-2 (Dianellin). Fig. S4. II: 1H, 13C, DEPT and HRMS of KFP-1 (Knipholone). Table S1. Antimalarial activity of the phenolic fractions of Kniphofia folosia in mice infected with Plasmodium berghei. Table S2. Prediction of partition coefficient Log P, aqueous solubility Log S and partition coefficient for partially dissociated compounds Log D of the compounds. Table S3. Docking result of compounds on the crystal structure of plasmepsin II (4cku) and plasmodium falciparum l-lactate dehydrogenase (pfLDH) (PDB 1ldg). Table S4. Acute oral toxicity results of dianellin. Fig. S4. Microscope slide photos of the negative control groups (A and B), dianellin and knipholone treated groups (C-E) and positive control group (F). [file 12936_2020_3552_MOESM1_ESM.docx]

**Extracted with 80% MeOH by maceration**

**80% MeOH extract**

**390 g powdered rhizomes of *Kniphofia foliosa***

**Filtered, concentrated under vacuum, and freeze dried**

**Dried 80% MeOH extract * (Yield=6.6%)**

**extract**

**Dissolved in 5% KOH soluti**

***= *In vivo* active**

**Crude Phenol fraction-1* (CHCl_3_ fraction)**

**CHCl_3_ layer**

**Crude Phenol fraction-2* (Precipitate)**

**Aqueous fraction**

**Acidified with 2% HCl**

**Aqueous layer (Basic)**

**Partitioned with equal volume of CHCl_3_**

**Partitioned with CHCl_3_**

**Concentrated and applied on PTLC [Toluene: EtOAc (4:1)]**

**KFP-1 (Knipholone )― 18 mg (Red solid, ED_50_=81.25 mg/kg)**

**Fig. S1:** Isolation protocol of knipholone **(2)**

**Fractionated on flash silicagel column**

**80% MeOH rhizome extract of *Kniphofia foliosa* (3.2 g)**

**Fraction-4**

**Eluted with 100% CH3OH**

**Fraction-3**

**Eluted with 50% CHCl_3_:CH_3_OH**

**Fraction-2**

**Eluted with 50% CHCl_3_:CH_3_OH**

**Fraction-1**

**Eluted with 100% CHCl_3_**

**Concentrated under vacuum, Suspended in H_2_O and then filtered**

**The water filtrate**

**Marc**

**Freeze dried**

**Viscous solid**

**Passed through RPFCC (Solid phase extraction)**

**Fraction-2 (Eluted with 100% MeOH)**

**Fraction-1 (Eluted with 100% water)**

**Freeze dried**

**Soft yellowish solid**

**Applied on PTLC [BAW: EtOAc (1:1)]**

**Yellow solid (Faint blush on analytical TLC)**

**Purified by RPFCC**

**YKFM-2 (Dianellin)** **― 10 mg (Red solid, ED_50_=92.31 mg/kg)**

**Fig. S2:** Isolation protocol of dianellin (**1**)

# Fig. S3 I: ^1^H, ^13^C, DEPT and HRMS of YKFM-2 (Dianellin)

Ia: ^1^H NMR spectrum of YKFM-2, methanol-*d*_4_


1.
2. Ib: ^13^C NMR spectrum of YKFM-2, methanol-*d*_4_


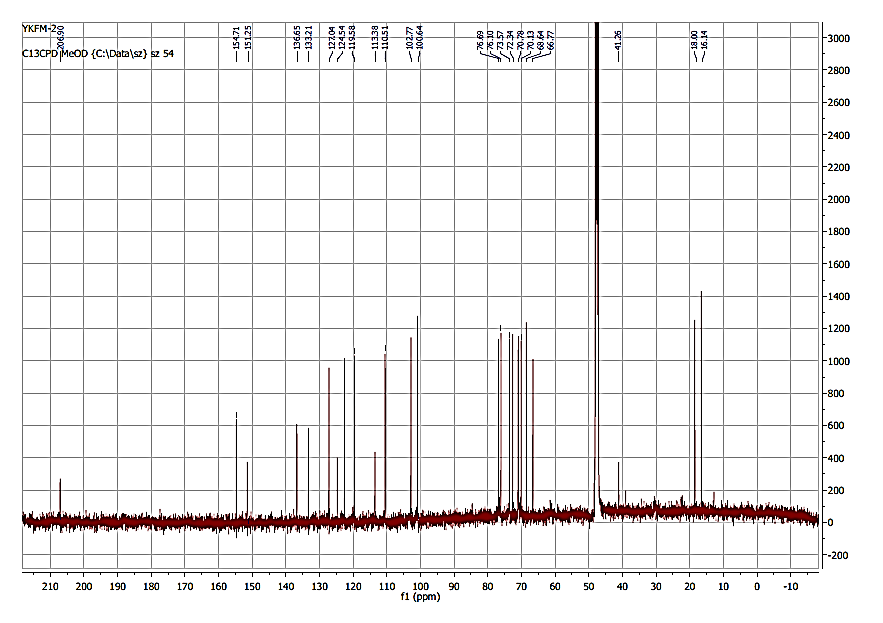


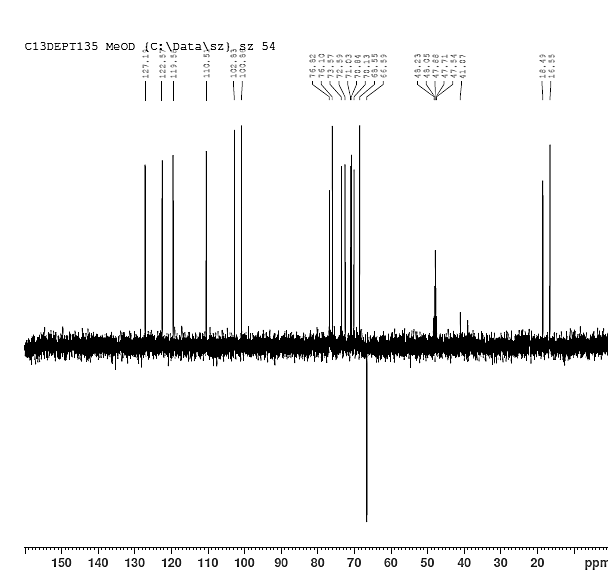
Ic: DEPT spectrum of YKFM-2, methanol-*d*_4_

Id: HRMS spectrum of YKFM-2


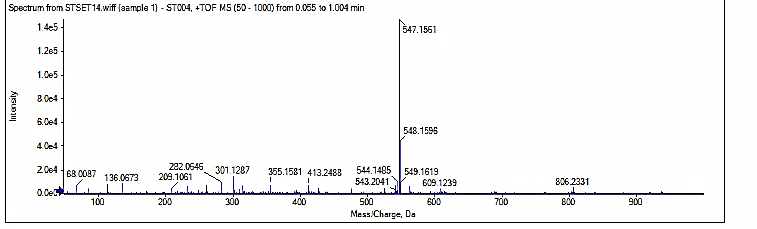


#

# Fig. S4 II: ^1^H, ^13^C, DEPT and HRMS of KFP-1 (Knipholone)

IIa: ^1^H NMR spectrum of KFP-1, chloroform-*d*


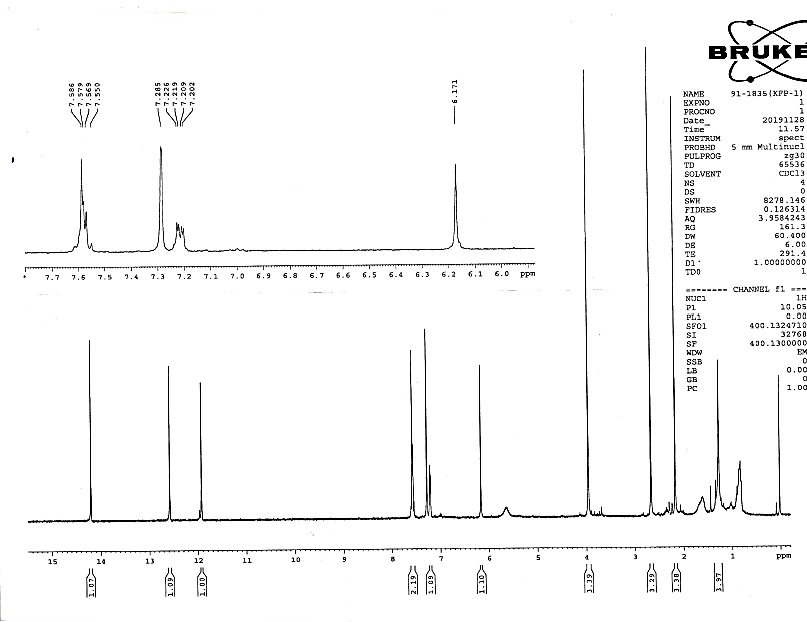


IIb: ^13^C NMR spectrum of KFP-1, chloroform-*d*


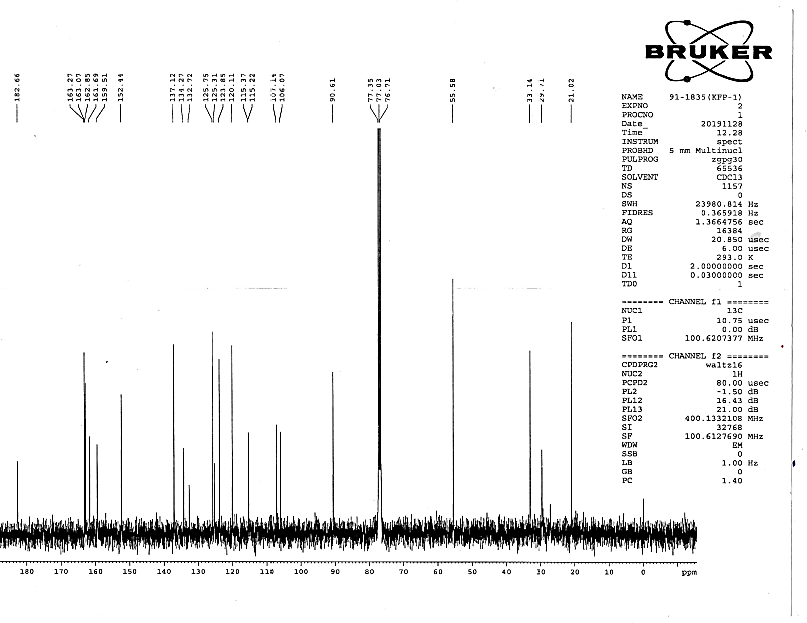


IIc: DEPT spectrum of KFP-1, chloroform-*d*


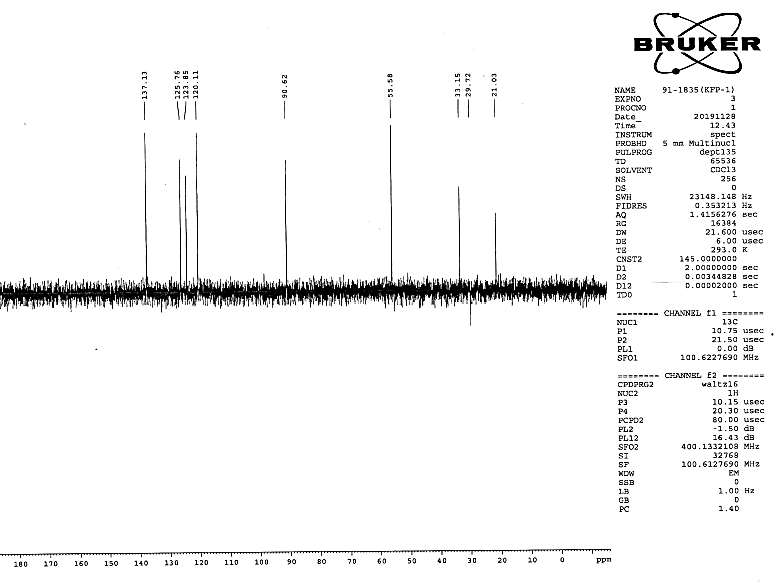


IId: Negative mode HRMS spectrum of KPF-1


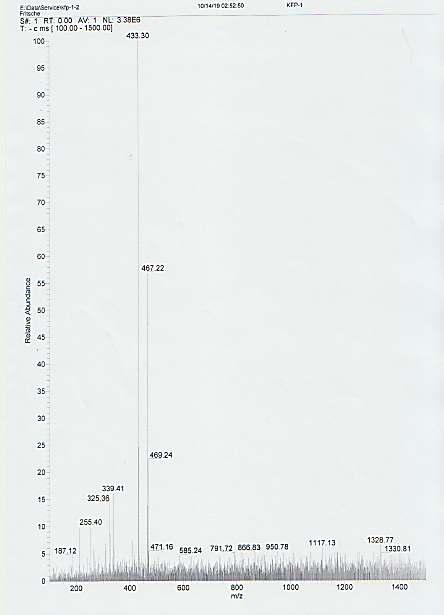


IId: Positive mode HRMS spectrum of KPF-1


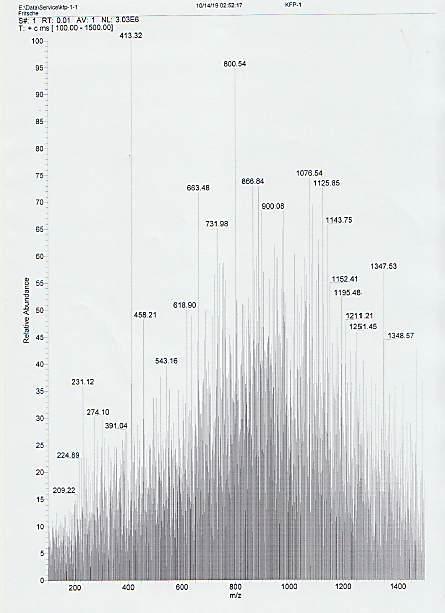


**Supplementary Tables**

**Table S1**. Antimalarial activity of the phenolic fractions of *Kniphofia folosia* in mice infected with *Plasmodium berghei.*

| **Test substances** | **Dose (mg/kg/day)** | **Percent Parasitaemia** | **Percent suppression** | **Mean survival time**  **(in days)** |
| --- | --- | --- | --- | --- |
| Vehicle2 | 0.2ml | 38.25400±0.61845 | 0.0000 | 6.0000±0.70711 |
| P-F1 | 100mg | 26.5120±1.24295 | 30.3500^a*i*^ | 7.8000±1.30384^i*^ |
| P-F1 | 200mg | 24.1000±1.27770 | 37.0700^a*i*^ | 8.2000±1.64317^i*^ |
| P-F1 | 400mg | 20.6240±1.56333 | 46.3200^a*i*^ | 8.2000±1.09545^i*^ |
| Vehicle1 | 0.2ml | 35.9860±1.22034 | 0.0000 | 6.0000±.31623 |
| P-F2 | 100mg | 26.2020±1.35847 | 27.1900^a**h**i*^ | 8.2000±1.09545^i*^ |
| P-F2 | 200mg | 21.9360±1.75275 | 39.0400^a*i*^ | 7.6000±1.14018^i*^ |
| P-F2 | 400mg | 18.8820±.98484 | 47.5300^a*f**i*^ | 8.2000±0.83666^i*^ |
| Chloroquine | 25mg | .0140±.00600 | 99.8000^a* b*c*d*e*f*g*h*^ | 27.2000±.58310^a*b*c*d*e*f*g*h*^ |

Values are presented as mean ± SEM; n =5; a = compared to vehicle-2 (1% tween 80), b = compared to P-F1 100mg, c = compared to P-F1 200mg, d = compared to P-F1 400mg, e = compared to vehicle-1 (distilled water), f = compared to P-F2 100mg, g = compared to P-F2 200mg, h = compared to P-F2 400mg, i= compared to chloroquine, * (p < 0.001); **(p < 0.01); PF-1= phenol fraction-1 of *K. folosia*, PF-2= phenol fraction-2 of *K. folosia*; numbers refer to doses in mg/kg/day.

**Table S2.** Prediction of partition coefficient Log P, aqueous solubility Log S and partition coefficient for partially dissociated compounds Log D of the compounds.

| **Compounds** | **Structure** | **Log P** | **Log S** | **Log D** |
| --- | --- | --- | --- | --- |
| Knipholone ‒ ZINC000004098683 |  | 3.47 | 0 | 3.253 |
| Dianellin |  | -2.75 | 3.391 | -1.468 |
| Chloroquine |  | 2.64 | 2.780 | 2.287 |
| Artemisinin |  | 2.64 | 2.232 | 3.042 |
| P2FE-400 |  | 4.74 | 0.439 | 1.954 |

**Table S3.** Docking result of compounds on the crystal structure of plasmepsin II (4cku) and plasmodium falciparum l-lactate dehydrogenase (pfLDH) (PDB 1ldg).

| **Plasmepsin II (4cku)** | | **Plasmodium falciparum l-lactate dehydrogenase (pfLDH) (PDB 1ldg).** | |
| --- | --- | --- | --- |
| **Compounds** | **HYDE score**  **∆G_HYDE_ (kj/mol)** | **Compounds** | **HYDE score**  **∆G_HYDE_ (kj/mol)** |
| P2FE-400 | -38.3 | Knipholone ‒ ZINC000004098683 | -29.1 |
| Chloroquine | **-**19.7 | Chloroquine | -24.7 |
| Artemisinin | -10.1 | Artemisinin | -10.4 |
| Knipholone ‒ ZINC000004098683 | -6.5 |  |  |
| Dianellin | -4.2 |  |  |

**Table S4.** Acute oral toxicity results of dianellin

| **Mice code** |  | **Follow up days** | | | **Mean weight and mean survival** |
| --- | --- | --- | --- | --- | --- |
|  |  | **Day0** | **Day7** | **Day14** |  |
| 01 | Dose administrated at Day0 (2/11/2020) | 2000mg/kg | - | - | - |
|  | Weight in gram | 23.52 | 27.13 | 29.97 | 26.87 |
|  | Sign of toxicity | Immediate signs ruffled fur and slight sleepiness; loss of appetite | None | None | - |
|  | Survival | No death | No death | No death | 15 |
| 02 | Dose administrated at Day0(3/11/2020) | 2000mg/kg | - | - | - |
|  | Weight in gram | 24.93 | 28.35 | 32.85 | 28.71 |
|  | Sign of toxicity | Immediate sign of ruffled fur; and loss of appetite | None | None | - |
|  | Survival | No death | No death | No death | 15 |
| 03 | Dose administrated at Do(4/11/2020) | 2000mg/kg | - | - | - |
|  | Weight in gram | 21.15 | 26.72 | 29.49 | 25.77 |
|  | Sign of toxicity | Immediate sign of ruffled fur; and loss of appetite | None | None | - |
|  | Survival | No death | No death | No death | 15 |
| 04 | Dose administrated at Day0(5/11/2020) | 2000mg/kg | - | - | - |
|  | Weight in gram | 24.40 | 25.55 | 30.11 | 26.67 |
|  | Sign of toxicity | Immediate sign of ruffled fur | None | None | - |
|  | Survival | No death | No death | No death | 15 |
| 05 | Dose administrated at Day0(6/11/2020) | 2000mg/kg | - | - | - |
|  | Weight in gram | 22.12 | 29.74 | 28.9 | 26.92 |
|  | Sign of toxicity | Immediate sign of ruffled fur; and loss of appetite | None | None | - |
|  | Survival | No death | No death | No death | 15 |


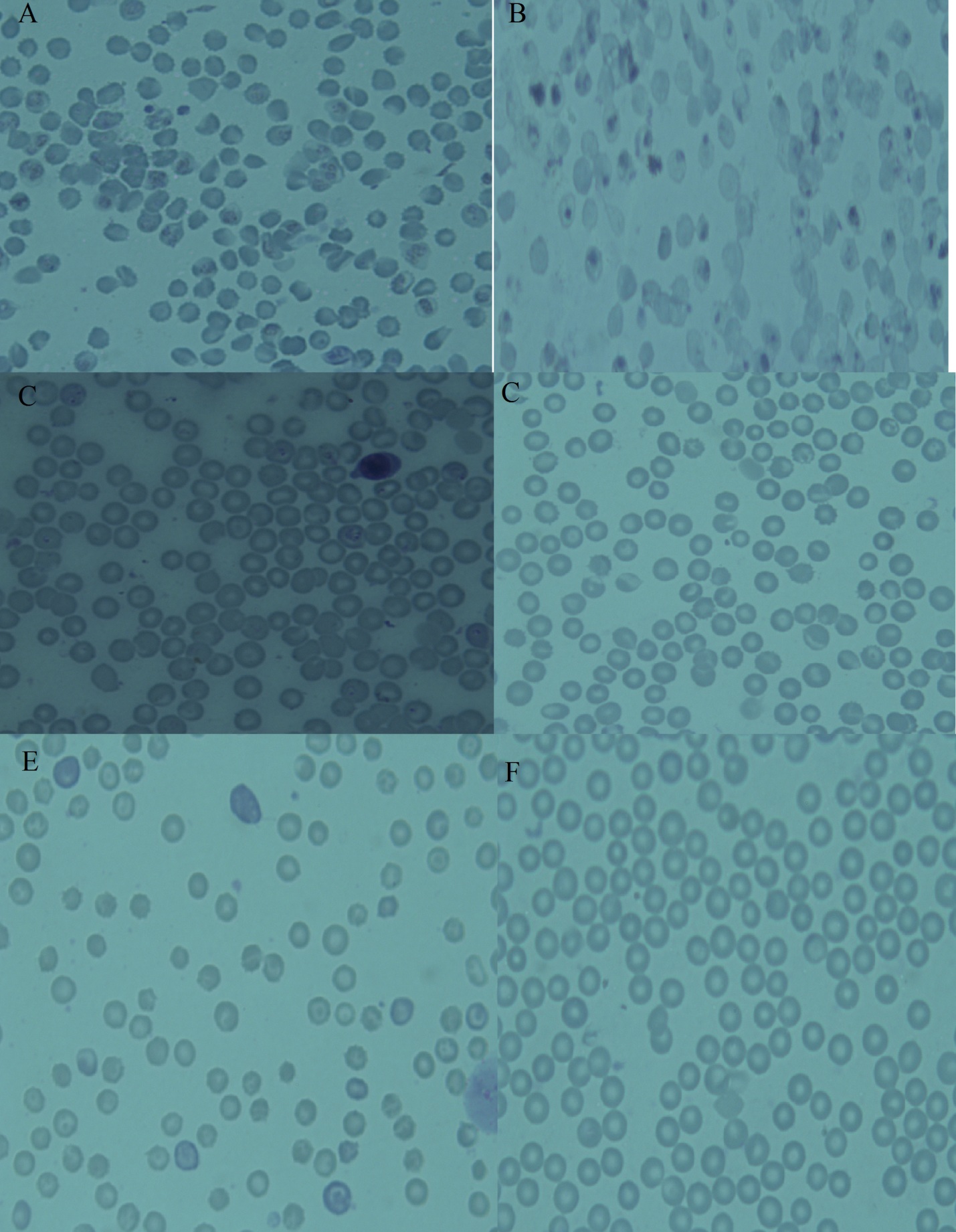
 **Fig. S4:** Microscope slide photos of the negative control groups (A and B), dianellin and knipholone treated groups (C-E) and positive control group (F).
